# Alcoholic cardiomyopathy: The result of dosage and individual predisposition

**DOI:** 10.1007/s00059-016-4469-6

**Published:** 2016-08-31

**Authors:** B. Maisch

**Affiliations:** Herz- und Gefäßzentrum Marburg (HGZ) und Philipps Universität Marburg, Feldbergstr. 45, 35043 Marburg, Deutschland

**Keywords:** Atrial fibrillation, Beriberi, Cirrhotic cardiomyopathy, Hypertension, Myocarditis, Vorhofflimmern, Beriberi, Zirrhosebedingte Kardiomyopathie, Hochdruck, Myokarditis

## Abstract

The individual amount of alcohol consumed acutely or chronically decides on harm or benefit to a person’s health. Available data suggest that one to two drinks in men and one drink in women will benefit the cardiovascular system over time, one drink being 17.6 ml 100 % alcohol. Moderate drinking can reduce the incidence and mortality of coronary artery disease, heart failure, diabetes, ischemic and hemorrhagic stroke. More than this amount can lead to alcoholic cardiomyopathy, which is defined as alcohol toxicity to the heart muscle itself by ethanol and its metabolites. Historical examples of interest are the Munich beer heart and the Tübingen wine heart. Associated with chronic alcohol abuse but having different etiologies are beriberi heart disease (vitamin B1 deficiency) and cardiac cirrhosis as hyperdynamic cardiomyopathies, arsenic poising in the Manchester beer epidemic, and cobalt intoxication in Quebec beer drinker’s disease. Chronic heavy alcohol abuse will also increase blood pressure and cause a downregulation of the immune system that could lead to increased susceptibility to infections, which in turn could add to the development of heart failure. Myocardial tissue analysis resembles idiopathic cardiomyopathy or chronic myocarditis. In the diagnostic work-up of alcoholic cardiomyopathy, the confirmation of alcohol abuse by carbohydrate deficient transferrin (CDT) and increased liver enzymes, and the involvement of the heart by markers of heart failure (e.g., NT-proBNP) and of necrosis (e.g., troponins or CKMb) is mandatory. Treatment of alcoholic cardiomyopathy consists of alcohol abstinence and heart failure medication.

## Zusammenfassung

Die individuelle Menge akut oder chronisch getrunkenen Alkohols ist für den gesundheitlichen Schaden oder Nutzen entscheidend. Grenzdosen für Männer sollten 1–2 Getränke à 17,6 ml reinen Alkohols sein, für Frauen ein Getränk. Mäßiger Alkoholkonsum kann die Inzidenz und Mortalität von koronarer Herzerkrankung, Herzinsuffizienz, Diabetes mellitus, ischämischem und hämorrhagischem Schlaganfall vermindern. Größere Mengen Alkohol führen zur alkoholischen Kardiomyopathie, bedingt durch die Toxizität von C_2_H_5_OH und seinen Metaboliten auf den Herzmuskel. Historische Beispiele sind das Münchener Bier- und das Tübinger Weinherz. Nicht auf die reine Alkoholwirkung zurückzuführen sind aufgrund ihrer andersartigen Ätiologie das Beriberi-Herz bei Mangel an Thiamin (Vitamin B1) und die kardial mitbedingte Leberzirrhose als hyperdyname Kardiomyopathien, die Arsenvergiftung bei der Manchester Biertrinkerepidemie und die nach Quebec benannte Bierherzerkrankung infolge einer Kobaltintoxikation. Chronischer Alkoholabusus erhöht den Blutdruck und verursacht eine Downregulation des Immunsystems mit erhöhter Infektanfälligkeit. Histologisch ist die alkoholische nicht von der idiopathischen Kardiomyopathie und einer chronischen Myokarditis zu unterscheiden. Alkoholabusus wird durch Bestimmung des carbohydratdefizienten Transferrins (CDT) und erhöhter Leberenzyme bestätigt, die Herzinsuffizienz durch Biomarker wie NT-proBNP und die Myozytolyse durch Troponine und CKMb. Die Behandlung der alkoholischen Kardiomyopathie besteht in der Alkoholabstinenz und medikamentöser Therapie der Herzinsuffizienz.

## Alcoholism—use and abuse

According to the definition of the World Health Organization (WHO), alcoholism is subgrouped in two categories: alcohol abuse and alcohol dependence [1]. This corresponds roughly with the concept of the American Psychiatric Association [2, 3]. Alcohol abuse describes the psychological dependence on ethanol for adequate functioning together with occasional heavy consumption, while alcohol dependence is defined as an increased alcohol tolerance together with physical symptoms upon withdrawal. In Western countries it is estimated that up to 10 % of the adult population suffers from alcoholism [4]. The highest prevalence is detected in the third to fifth decade of life, and alcoholism is seen in all races, ethnic groups, and socioeconomic strata.

Germany with a total population of 81 million inhabitants is a permissive society with respect to the drinking of alcohol. Alcohol consumption is part of the local culture. About 40 million individuals drink alcohol. The per capita alcohol consumption of 9.7 l pure ethanol and the early onset of regular or episodic intensive drinking among young people in Germany consequently leads to high alcohol-related morbidity and mortality [5].

More than 1.8 million individuals in Germany with a total population of 81 million inhabitants are alcohol dependant. For an additional 1.6 million persons the use of alcohol is harmful [6, 7]. In a world-wide setting, alcohol use disorders show similarities in developed countries, where alcohol is cheap and readily available [8]. The many complications of alcohol use and abuse are both mental and physical—in particular, gastrointestinal [9], neurological [10, 11], and cardiological [12, 13]. The relationship of alcohol with heart disease or dementia is complicated by the fact that moderate alcohol consumption was shown not only to be detrimental but to a certain degree also protective against cardiovascular disease [14] or to cognitive function in predementia.

We reviewed the effects of ethanol on the cardiovascular system in 1996 [15], including aspects of inflammation [16], rhythm disturbances [17], and hypertension [18]. In 2001 we updated the data on the ambivalent relationship between alcohol and the heart [19] and in 2008 added new evidence on a larger cohort of patients with different forms of cardiomyopathy and increased alcohol intake from the German competence network on heart failure [20].

This review revisits our past and deals with our current thinking on the epidemiology, pathophysiology, clinical characteristics, and treatments available for alcoholic cardiomyopathy.

## Methods

This review assembles and selects pertinent literature on the ambivalent relationship of ethanol and the cardiovascular system, including guidelines, meta-analyses, Cochrane reviews, original contributions, and data from the Marburg Cardiomyopathy registry.

Drinks as measures of alcohol are often given in ounces (oz), whereby 1 oz equals 28.35 g or 29.57 ml.

Examples for 100 % alcohol in ml of ***one drink*** in consumed beverages are between 17.6 to 17.76 ml:

- **Beer:** 12 fluid ounces of 5 % beer = 355 ml fluid = 17.5 ml 100 % alcohol.
- **Wine:** 5 fluid ounces of 12 % wine = 148 ml fluid = 17.76 ml of 100 % alcohol.
- **Distilled spirits:** 1.5 fluid ounces of ~40 % liquor = 44 ml = 17.6 ml of 100 % alcohol.

## A historical perspective

For more than 3000 years, alcoholic beverages have been consumed in multiple societies through the centuries and cultures. The name alcohol is much younger than the many beverages containing it. Pulverized antimony was used as eye shadow by Egyptian women and named al-Kol. In the 16th century Paracelsus Theophrastus Bombastus from Hohenheim used this term for distilled liquor and called it alcohol [15]. The beneficial cardiovascular effects of alcohol have been appreciated, e. g., in medieval times, when people took advantage of the vasodilating properties of alcohol to treat angina pectoris or heart failure. So Hildegard von Bingen (1098–1179), one of the most prominent mysticians of her time, recommended her heart wine as a universal remedy. One liter of wine was cooked for 4 min with 10 fresh parsley stems, 1 spoon of vinegar, and 300 g honey and then filtered [11]. This recipe is still in use today.

**Fig. 1:**
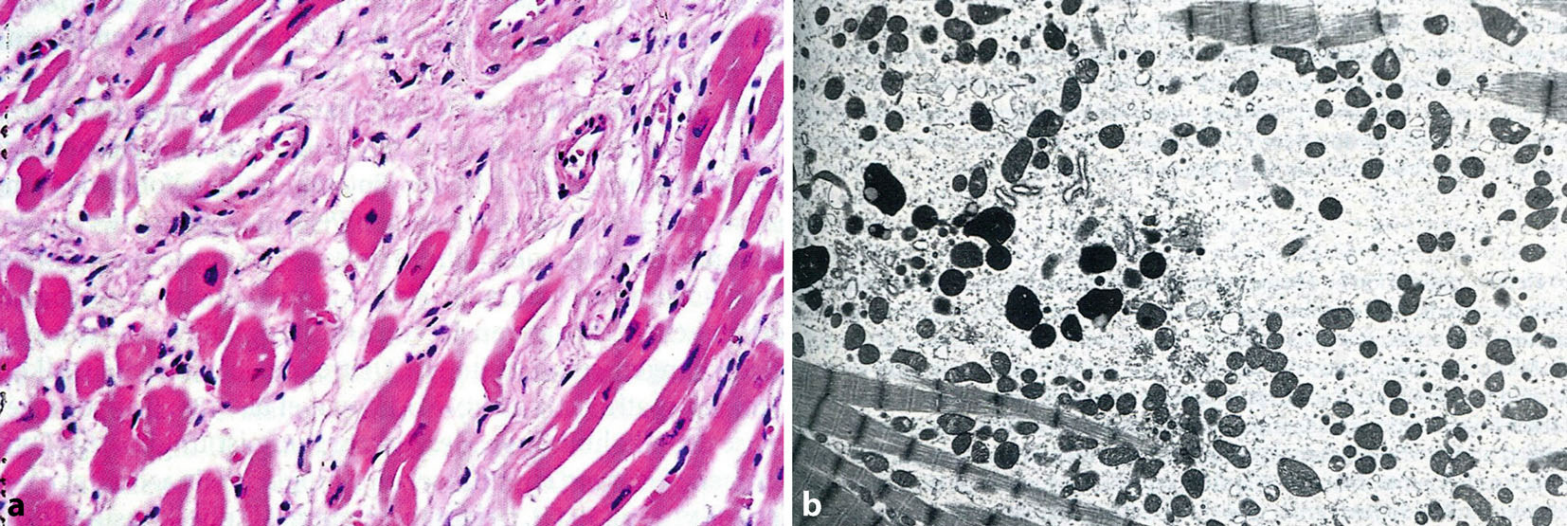
**a** Left ventricle from a 49-year-old man with chronic alcohol abuse. Myofibers show partly hypertrophy and atrophy. Fibrosis is present as reparative interstitial and perivascular fibrosis. HE ×320. **b** Electron microscopy of an endomyocardial septal biopsy from a patient with alcoholic cardiomyopathy demonstrating myofibrillar reduction and variable mitochondriae in size but increased in number. ×2190. (With kind permission from H. Frenzel and B. Schwartzkopff [22])

Over the centuries “the good and the bad” of alcohol were evaluated clinically and scientifically. As early as 1855, Wood incriminated alcohol as a cause of heart failure. In 1861, Friedrich reported idiopathic hypertrophy as associated with alcoholism. In 1873, Walshe described myocardial cirrhosis in alcoholics, which includes a spectrum of hepatic derangements that occur in the setting of right-sided heart failure. Conversely cirrhosis (fibrosis) was found both in heart and liver. High cardiac output in patients with liver cirrhosis may have contributed to this cardiomyopathy in a vicious circle. The term “wine heart” (Tübinger Weinherz) originated in 1877 by Münzinger [21], a German pathologist at Tübingen university. This entity we would call nowadays “alcoholic cardiomyopathy” with histologic features of dilatation, myofibrillar necrosis and fibrosis (Fig. 1a), and ultrastructural changes such as reduction of myofibrils and mitochondriosis in a great variability of size and form (Fig. 1b; [22]).

**Fig. 2:**
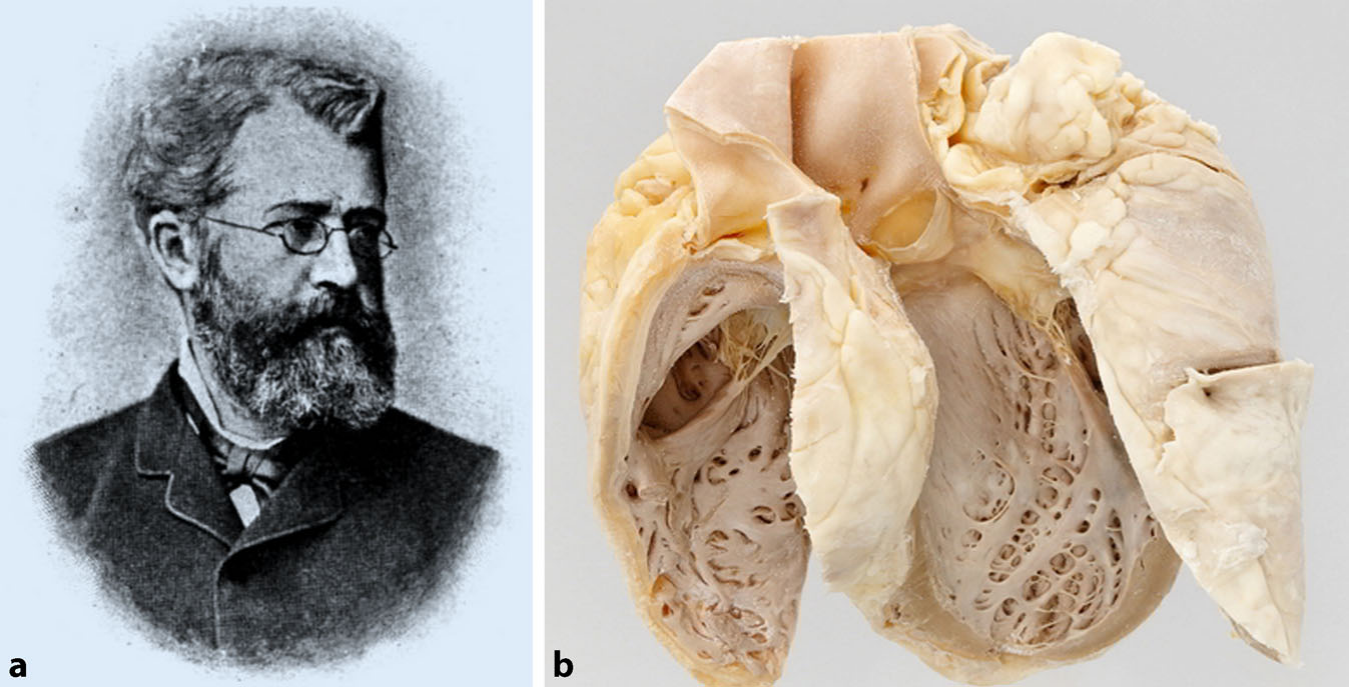
**a** Otto von Bollinger. (© de.wikipedia.org). **b** Munich beer heart. (© Philipp Mansmann in http://www.bayerische-staatszeitung.de/staatszeitung/kultur/detailansicht-kultur/artikel/bierherz.html)

In Munich, the annual consumption of beer reached 245 l per capita and year in the last quarter of the 19th century. In 1884, the pathologist and veterinarian Otto von Bollinger (Fig. 2a) described the “Munich beer heart” with fibrosis, hypertrophy, and fatty degeneration in postmortem cardiac tissue of alcoholics who consumed an estimated average of 432 liters of beer per year (Fig. 2b; [23]). At that time every 10th necropsy in men at the Munich pathology institute named cardiac dilatation and fatty degeneration as “Bierherz” being its underlying cause. For comparison, the mean annual beer consumption in Bavaria is nowadays estimated to be 145 l and in the rest of Germany around 100 l beer per person and year [24].

In 1887, Maguire reported on 2 patients with severe alcohol consumption who benefitted from abstinence. He suggested that alcohol was poisoning the heart. In 1890, Strümpell listed alcoholism as a cause of cardiac dilatation and hypertrophy, as did Sir William Osler in 1892 in his textbook *Principles and Practices of Medicine*. In 1893, Graham Steell, well known for the Graham Steell murmur due to pulmonary regurgitation in pulmonary hypertension or in mitral stenosis, reported 25 cases in whom he recognized alcoholism as one of the causes of muscle failure of the heart. He found it “a comparatively common one” [25]. In his 1906 textbook *The Study of the Pulse*, William MacKenzie described cases of heart failure attributed to alcohol and first used the term “alcoholic heart disease” [26].

In his 1972 review article, Bridgen was the first to introduce the term alcoholic cardiomyopathy [27].

## Nutritional causes of “alcoholic” cardiomyopathy

### Beriberi heart disease

Thiamine deficiency is common feature in a malnourished and/or alcoholic population. Thus, the concept of beriberi heart disease dominated thinking about alcohol and the heart for decades and caused many to doubt that alcohol was actually cardiotoxic [28]. But vitamin B1 (thiamine) deficiency is accompanied by an elevated cardiac output and diminished peripheral vascular resistance [29, 30]. According to its central hemodynamics, it can be classified as *hyperdynamic cardiomyopathy* or high output failure with a cardiac output >8 l/min or a cardiac index >3.9 l/min/m^2^ [31, 32]. In contrast, alcoholic cardiomyopathy is characterized by a low cardiac output, associated with systemic vasoconstriction [4]. However, the high output state can lead to cardiac dilation, thus, representing a characteristic subentity of cardiomyopathy different from low output dilated cardiomyopathy. Therefore, thiamine deficiency per se is just a historical nutritional anomaly in the history of alcoholic cardiomyopathy.

### Manchester arsenic-in-beer epidemic

In 1900, the Manchester arsenic-in-beer epidemic was a serious food poisoning outbreak affecting several thousand people across the North-West and Midlands of England, with many cases proving fatal. The arsenic had come from the glucose for which sulphuric acid was used in the sugar production process of a company in Leeds. Brewers had been using this sugar, thus, unknowingly poisoning the beer and as a result their customers for many years even prior to the epidemic [33]. Arsenic poising caused a multisystem disease in over 6000 cases with more than 70 deaths [34]. The syndrome included the usual signs and symptoms of arsenic poisoning, with skin, nervous system, and gastrointestinal manifestations. Unusual in arsenic poisoning, but especially prominent in this epidemic, were the cardiovascular findings. In his clinical description, Ernest Reynolds wrote that “cases were associated with so much heart failure and so little pigmentation that they were diagnosed as beri-beri …”. He also found that “undoubtedly the principal cause of death has been cardiac failure. In postmortem examinations, the only prominent signs were the interstitial nephritis and the dilated flabby heart” (p. 169, [35]). This outbreak had been the first known trace metal cardiotoxic syndrome.

In 2013, the issue of arsenic in beer and wine was again prominent, when Mehmet Coelhan, a researcher at the Weihenstephan research center at the Technical University of Munich, reported at a meeting of the American Chemical Society that many of the nearly 360 beers tested in Germany had trace amounts of arsenic. The source was identified to be the filter of choice for wine and beer, i.e., diatomaceous earth [36]. The German word for it is Kieselguhr, a beige powder made up of the skeletons of diatoms. The trace amounts of arsenic have not been comparable to the arsenic-in-beer endemic in Manchester but may still reach up to 10-times the amount admitted for arsenic in drinking water in the European Union and the US.

### Quebec‘s beer drinker disease

In the mid-1960s, another unexpected heart failure epidemic among chronic, heavy beer drinkers occurred in two cities in the USA, in Quebec, Canada, and in Belgium. It was characterized by congestive heart failure, pericardial effusion, and an elevated hemoglobin concentration. The explanation proved to be the addition of small amounts of cobalt chloride. Cobalt was used as a foam stabilizer by certain breweries in Canada and in the USA. In 1966 McDermott et al. [37] described the syndrome as myocardosis with heart failure, Kestelott et al. [38] added pericardial involvement and named it alcoholic pericardiomyopathy, and Morin and Daniel [39] in Quebec tracked down the etiology to cobalt intoxication to what become known as Quebec beer-drinkers cardiomyopathy. Human pathology was first described by Bonefant et al. [40]. Animal models investigated ultrastructure [41] and treatment e. g. by selenium [42]. Removal of the cobalt additive ended the epidemic in all locations. Cobalt poisoning and alcohol together acted synergistically in these patients. As the syndrome could be attributed to the toxicity of this trace element, the additive was prohibited thereafter.

Not alcohol but cobalt itself recently caused severe heart failure in a 55-year-old man, who was referred to the university hospital in Marburg to rule out coronary artery disease as the cause of his heart failure. He had become almost deaf and blind, with fever of unknown cause, hypothyroidism, and enlarged lymph nodes. Both his hips had been replaced, the left side by a CoCrMo Protasul metal prosthesis. Remembering a similar case in an episode of the TV series *Dr. House*, the team of J. Schäfer suspected cobalt intoxication as the cause of heart failure, which clinically mimicked Quebec‘s beer drinker disease [43]. One should note, however, that cobalt is needed in minute amounts of 0.0003 mg/day in vitamin B12 (cobalamine) to avoid megaloblastic anemia.

## Cardiac cirrhosis or cirrhotic cardiomyopathy

The heart and liver interact in several different ways. Acute or chronic right heart failure leads to elevation of liver enzymes most likely due to liver congestion, whereas  cirrhosis due to cardiac disease is infrequent. Chronic liver disease such as cirrhosis may in turn affect the heart and the whole cardiovascular system, leading to a syndrome named cirrhotic cardiomyopathy (CCM). Thus, CCM has been introduced as an new entity separate of the cirrhosis etiology. Increased cardiac output due to hyperdynamic circulation, left ventricular dysfunction (systolic and diastolic), and certain electrophysiological abnormal findings are pathophysiological features of the disease. The underlying mechanisms might include the impaired β‑receptor and calcium signaling, altered cardiomyocyte membrane physiology, elevated sympathetic nervous tone and increased activity of vasodilatory pathways [44]. In pathophysiological terms, heart failure in liver cirrhosis belongs to the hyperdynamic cardiomyopathies.

## Hypertension

As early as in 1915, Lian [45] reported in middle-aged French servicemen during the first world war that heavy drinking could lead to hypertension. It took almost 60 years before further attention was paid to the complex interaction between the heart and the peripheral vasculature in various cross-sectional and prospective epidemiologic studies, which have empirically confirmed this early report. One is aware today that alcohol may cause an acute but transient vasodilation, which may lead to an initial fall in blood pressure probably mediated by the atrial natriuretic peptide (ANP) [46]. But also short- and long-term pressor effects mediated by the renin–aldosterone system and plasma vasopressin have been described [47, 48].

The long-term hypertensive effect of alcohol has been confirmed in many studies [49–52]. Remarkably, alcohol also interacts with brain stem receptors and exerts thereby central hypertensive effects [18]. The apparent threshold amount of drinking associated with higher blood pressure is approximately 3 drinks/day. Most studies show no increase in blood pressure with lighter drinking; several show an unexplained J‑shaped curve in women with lowest blood pressures in lighter drinkers. There seems to be independence from adiposity, salt intake, education, smoking, beverage type (wine, liquor, or beer), and several other potential confounders.

Clinical observation confirmed that several days to weeks of drinking show higher and weeks of abstinence lower pressures. Alcohol intake may also interfere with the drug and dietary treatment of hypertension. This altogether supports a causal relationship between alcohol consumption and a hypertensive state.

## Alcoholic cardiomyopathy: Cytotoxicity of alcohol on heart muscle

The 1989 landmark report of Urbano-Marquez et al. [53] showed a clear relation of lifetime alcohol consumption to structural and functional myocardial and skeletal muscle abnormalities in alcoholics. The amount of consumed alcohol was large—the equivalent of >80 g alcohol/day for 20 years. Further evidence came from data on acute alcohol effects [54] and from clinical observation [55–57].

In 1996, cardiomyopathies were defined as diseases ”affecting the myocardium with associated cardiac dysfunction“ [58] and primary and secondary forms were distinguished in this context. After consumption of large quantities of alcohol over years the clinical picture of heavy alcohol drinkers could be indistinguishable from other forms of dilated or familial cardiomyopathy. Alcohol is still suspected to be the major cause or contributory factor of secondary nonischemic dilated cardiomyopathy being involved in up to one third of all cases of dilated cardiomyopathy [59–61]. In alcoholic cardiomyopathy, dilation and impaired contraction of the left or both ventricles is observed [4]. Left ventricular enddiastolic diameters are increased compared to age- and weight-matched controls [62], the left ventricular mass index is increased [63], and the left ventricular ejection fraction is well below normal (<45 %). Thus, the diagnosis of alcoholic cardiomyopathy is still based on the coincidence of heavy alcohol consumption and a global myocardial dysfunction, which cannot be explained by any other underlying myocardial disease [64]. However, the prevalence of alcoholic cardiomyopathy may be underestimated, as autopsy findings reveal pathologic changes of the heart in individuals with no clinical symptoms [65], when analyzing in large cross-sectional studies.

Further evidence suggests that not only ethanol but also the first metabolite acetaldehyde may directly interfere with cardiac and skeletal muscle homeostasis [53, 66]. In vitro studies have further elucidated the direct effect of ethanol on electromechanical coupling, indicating a decrease in myofilament–calcium sensitivity during alcohol consumption, changes in the transmembrane action potential, the amplitude of the cytosolic calcium transients, and the shortening of the action potential duration [67–71]. Isolated cardiomyocytes of alcohol-fed rats did not maintain ATP levels upon energy demand due to an inadequate increase in mitochondrial ATP-synthase activity, which led altogether to further myocyte loss [72, 73]. Ultrastructural disarray of the contractile apparatus [74] is associated with a depressed myofibrillar and sarcoplasmic protein synthesis in cardiac muscle after ethanol exposure [75–77]. This reduces contractile cardiac filaments with subsequent negative inotropic effects on heart contractility [78, 79]. An apoptotic effect of ethanol on cardiac muscle has also been described, which could be counteracted by insulin-like growth factor (IGF)-I [80] and confirmed in later studies [81, 82]. In a study in rats that were fed with two different doses of alcohol (5 mM [low alcohol], 100 mM [high alcohol] or in pair-fed nonalcohol controls for 4–5 months), caspase-3 activity as putative marker of apoptosis was decreased in the low alcohol diet, which went along with increased or normal contractility, whereas high doses of ethanol showed increased caspase activity, wall thinning, and a reduction of shortening velocity [83]. Of note, rats are a relatively alcohol resistant species.

## Alcohol and myocarditis

**Fig. 3:**
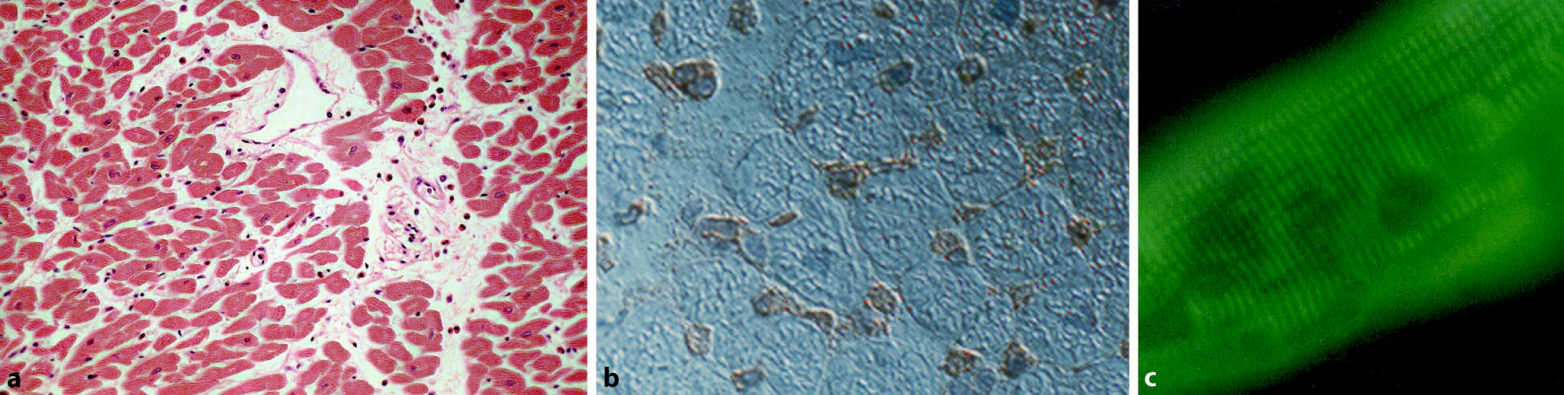
**a** Left ventricle (*LV*) biopsy of a 53-year-old individual with an alcohol consumption of >5 drinks/day for 32 years. Perivascular increase of leukocytes and fibrosis, myocytes in variable sizes with some myocytolysis. HE ×160. **b** LV biopsy of the 53-year-old alcoholic with increased ICAM (intercellular adhesion molecule) expression in capillaries and small vessels. ×320. **c** Circulating antimyosin antibodies in the 53-year-old patient with alcoholic abuse. Indirect immunofluorescent test. Titer 1:160 ×640

Alcohol abuse coinciding with myocarditis was reported in 1902 by McKenzie [26]. In endomyocardial biopsies of alcoholics up to 30 % of patients were found to exhibit sparse lymphocytic infiltrates with myocyte degeneration and focal necrosis and increased HLA (human leukocyte antigen) or ICAM (intercellular adhesion molecule) expression (Fig. 3; [16, 84]).

This may have to do with the susceptibility for infections due to a suppressed immune system in a compromised human host and also in experimental animal [85]. Ethanol can alter lymphocyte functions, inhibit neutrophil chemotaxis, and suppress the production of cytokines, which are involved in regulating acute inflammatory responses to infectious challenges [86–88]. Furthermore, autoimmunity and circulating autoantibodies seem to be associated in some patients with chronic alcohol consumption [16, 20, 84].

## Coronary artery disease and atherosclerosis

The beneficial heart wine as universal remedy in medieval ages by Hildegard von Bingen [11] found its later correlates in many observations at the beginning of modern medicine when coronary artery disease (CAD) and its risk factors and symptoms received more attention. Heberden [89] described angina so elegantly in 1786 and also added that ”considerable relief“ through ”wine and spirituous liquors“ could be expected. This observation led to the erroneous belief that alcohol is an immediate coronary vasodilator. Alcohol is not a direct coronary vasodilator [90]. Symptomatic relief of angina could be through the anesthetic effect of ethanol or through peripheral vasodilation, which could transiently reduce oxygen demand of the heart.

In 1819 the Irish physician Dr. Samuel Black, who had a special interest in angina pectoris described what is probably the first commentary pertinent to the ”French Paradox“ [91]. This refers to the finding in the last century that moderate alcohol consumption could be the reason for the relatively low cardiovascular disease incidence in wine-drinking regions [92]. Renaud and de Lorgeril [93] suggested that the inhibition of platelet reactivity by wine may be one explanation for protection from CAD in France. However, there was further evidence on this and other dietary mechanisms with the observation that France and Finland have similar intakes of cholesterol and saturated fat, but consumption of vegetables and vegetable oil containing monounsaturated and polyunsaturated fatty acids is greater in France than in Finland.

This inverse relation on mortality resembles in most population based studies a U- or J-shaped curve: Total abstinence has a slightly increased mortality when compared to low or moderate alcohol consumption. It is present in individuals with and without overt CAD, with diabetes, and with hypertension and has been underlined by a large number of studies [94, 95]. The cardioprotective effect of alcohol can be attributed to the increase in total high-density lipoproteins (HDL), and especially by an increase in subfractions HDL2 and HDL3, whereas established cardiovascular risk factors like low-density lipoproteins (LDL) or lipoprotein(a) are thought to be moderately decreased [96]. Moderate alcohol intake also exerts beneficial effects on the blood coagulation system. It leads to an increase of endogenous plasminogen activators [97], or a decrease in fibrinogen concentrations [98].

In the Caerphilly prospective heart disease study, platelet aggregation induced by adenosine diphosphate was also inhibited in subjects who drank alcohol [99]. Assessing differences between various forms of alcoholic beverages it should be noted that resveratrol leads in vitro to platelet inhibition in a dose-dependent manner [100] and has shown effects on all-cause mortality in a community-based study [101]. Polyphenols of red barrique wines and flavonoids have been shown to inhibit endothelin-1 synthase [102] and PDGF-induced vasoproliferation thus also contributing to cardiovascular protection [103].

## Signal transduction and beta-receptors

In alcoholic cardiomyopathy, similar to idiopathic dilated cardiomyopathy (DCM), beta 1‑adrenergic and muscarinic receptors are reduced in the myocardium itself and reduced responsiveness of the adenyl cyclase was shown, whereas catecholamine levels in the circulation may be elevated [104]. As a net effect, negative inotropism may result and contribute to heart failure.

## Arrhythmias and stroke

Acute effects of alcohol can result in rhythm disturbances. Since this happens often on weekends and holidays, Ettinger and Regan coined the term ”holiday heart syndrome“, when they described 32 habitual drinkers with an additional ingestion of ethanol prior to the arrhythmia [59, 105]. Atrial fibrillation was the commonest manifestation, which resolved with abstinence. In the Kaiser Permanente Study, atrial arrhythmias in 1322 persons reporting >6 drinks per day were compared to arrhythmias in 2644 matched light drinkers, showing a doubled relative risk for heavy drinkers [106]. Apart from direct cardiotoxicity, hypertension causing atrial stretch the arrhythmogenic potential of alcohol may come from the lowering the resting membrane potential [107] and the prolongation of conduction [108].

Studies of alcohol and stroke are complicated by the various contributing factors to stroke. Heavier drinkers are apparently at a higher risk of hemorrhagic stroke, whereas moderate drinking might be neutral or even result in a reduced risk of ischemic stroke.

## Clinical work-up for alcoholic cardiomyopathy

Habitual drinkers often hide their alcohol dependence fairly effectively. They may admit drinking at social events but not the abuse in the first contact. Patients with alcoholic cardiomyopathy, therefore, usually present with symptoms of heart failure, i. e., dyspnea, orthopnea, edema, nocturia, and tachycardia. Echocardiography may reveal a mild or severe depression of cardiac function and ejection fraction or even show hypertrophy in the beginning [109]. Heart failure symptoms may be due to early diastolic or to later systolic dysfunction. At later stages, due to atrial fibrillation, thrombi are not uncommon in the dilated atria. Mitral regurgitation is found in up to two thirds of cases [110]. Atrial fibrillation and supraventricular tachyarrhythmias are common findings in 15–20 % of patients [111], whereas ventricular tachycardias are rare [112]. On ECG, unspecific abnormalities like complete or incomplete left bundle branch block, atrioventricular conduction disturbances, alterations in the ST segment, and P wave changes can be found comparable to those in idiopathic DCM [113].

On endomyocardial biopsy, a discrimination between idiopathic, chronic inflammatory and alcoholic cardiomyopathy is virtually impossible since common features such as fibrosis, hypertrophy of cardiac myocytes, and alterations of nuclei are present at light microscopy in the alcoholic cardiomyopathy [114] as well as in chronic myocarditis according to the Dallas criteria [115] or the World Heart Federation/International Society and Federation of Cardiomyopathy (WHF/ISFC) definition of myocarditis [116]. Although the severity of histological alterations on endomyocardial biopsy correlates with the degree of heart failure in one of our studies, biopsy is not in common use for prognostic purposes [117]. Even the recovery after abstinence of alcohol is hard to predict based on morphometric evaluation of endomyocardial biopsies [118].

Cardiac MRI may be helpful in the differential diagnosis to hypertrophic cardiomyopathy, storage diseases, and inflammatory cardiomyopathy. For a comprehensive overview see Table 1 (combined data from [6, 8, 24, 28]).

**Table 1:** Clinical work-up in alcoholic cardiomyopathy

Work-up of	Criteria/findings
Cardiac symptoms	Fatigue, dyspnea, edema, nocturia, tachycardia
Noncardiac physical examination	Mental state (delirium tremens, depression, anxiety, psychosis) Neurology (cognitive decline, cerebellar degeneration, peripheral neuropathy, proximal myopathy) Respiratory function (aspiration pneumonitis, pneumonia, tuberculosis, smoking) Gastrointestinal tract (malnutrition, liver disease, pancreatic disease) Endocrine function: pseudo-Cushing’s syndrome, hypogonadism
ECG	Atrial fibrillation, complete or incomplete left or right bundle branch blocks, ST-segment and T‑wave alterations
Echocardiography	LV dilatation or hypertrophy, atrial dilatation, reduced shortening and ejection fraction, small pericardial effusion, mitral and tricuspid regurgitation, atrial thrombi in atrial fibrillation
Endomyocardial biopsy	Similar to dilated cardiomyopathy with myocyte hypertrophy or loss, reparative fibrosis, low grade leukocyte infiltration, variable, sometimes increase in Major Histocompatibility Complex(MHC) class I and II expression, immunoglobulin binding to sarcolemma and myosin; helpful in differential diagnosis of other forms of cardiomyopathies, theoretically suited for follow-up or improvement but not in common use for this purpose
Cardiac MRI	Helpful in ruling out other cardiomyopathies, e. g. hypertrophic cardiomyopathy, myocarditis, constrictive pericarditis
Cardiac CT	Only as noninvasive method to exclude coronary disease

## Laboratory findings

Measuring blood alcohol concentration in an *acute* intoxication gives baseline information but does not permit deductions to chronic misuse. Markers for *chronic *alcohol consumption rely on liver enzymes such as gamma-glutamyltransferase (GGT) [119], glutamic oxalacetic transaminase (GOT), and glutamic pyruvic transaminase (GPT). Elevations of the transaminases (GOT, GPT), especially a ratio of GOT/GPT higher than 2 might be indicative of alcoholic liver disease instead of liver disease from other etiologies [120, 121]. An excellent marker is carbohydrate deficient transferrin (CDT), which best detects chronic alcohol consumption alone [122, 123] or in combination with the other markers such as GGT [8, 124]. Markers such as ethyl sulphate, phosphatidyl ethanol, and fatty acid ethyl esters are not routinely done. For a comprehensive overview see Table 2 with combined data from [6, 8, 24, 28].

**Table 2:** Markers of alcoholism and cardiac involvement

Laboratory marker	Indicative for	Time to normalize	Monitor abstinence
Alcohol concentration	In acute alcohol intoxication	Hours	Yes
Mean corpuscular volume of red blood cells (MCV)	Increased	3 months	No
GGT, GOT, GPT, GOT/GPT ratio	Liver disease in patients with alcohol abuse	4 weeks	No
CDT (carbohydrate-deficient transferrin)	Chronic alcohol abuse	4 weeks	No
Ethyl glucuronide and ethyl sulphate	High-risk drinkers	2 days	Yes
Phosphatidyl ethanol	High-risk drinkers	4 weeks	No
NT-proBNP	Heart failure, helpful in follow-ups	Several weeks	No
Troponins, CKMB	Acute myocyte destruction	1–3 days	No

*MCV* mean corpuscular volume, *GGT* gamma-glutamytransferase, *GOT* glutamic oxalacetic transaminase, *GPT* glutamic pyruvic transaminase, *CDT* carbohydrate-deficient transferrin, *NT-proBNP* n-terminal pro brain natriuretic peptide, *CKMB* creatinin kinase, muscle, brain subunit

Biomarkers of heart failure such as NT-proBNP and of myocardial necrosis such as the troponins and CKMB indicate heart failure or myocytolysis.

## Is there an immediate risk of alcohol intake?

In a recent meta-analysis, Mostofsky et al. [125] analyzed if independent from habitual moderate or heavy alcohol consumption an immediate risks exists following alcohol intake. Data from 23 studies with 20,457 participants showed that even with moderate consumption an immediately higher cardiovascular risk was attenuated after 24 h. It then became protective for myocardial infarction and hemorrhagic stroke with a 30 % lower risk and protective against ischemic stroke within one week. In contrast, heavy alcohol drinking continued to be associated with higher cardiovascular risk in the following day (RR =1.3–2.3) and week (RR =2.25–6.2).

## Prognosis and treatment

Prognosis in individuals with low or moderate consumption up to one or two drinks per day in men and one drink in women is not different from people who do not drink at all. In CAD, diabetes, and stroke prevention the J‑type mortality curves even indicate some benefit apart from the social ”well-being“. In patients with chronic alcohol use disorders and severe heart failure prognosis is poor, since continued alcohol abuse results in refractory congestive heart failure. Death might also be sudden due to arrhythmias, heart conduction block, and systemic or pulmonary embolism. In these patients, only early and absolute abstinence of alcohol can reverse myocardial dysfunction [56, 57, 126] which in a historic study by McDonald and Burch was achieved with prolonged bedrest for several months without further access to alcoholic beverages. This was an excellent result long before ACE inhibitors or betablockers were available for heart failure treatment [57]. Mortality can otherwise reach 40–50 % within a 4–5 year period in the nonabstinent patients [127], whereas after withdrawal from alcohol hemodynamic and clinical improvement or at least a slower progression of disease compared to the idiopathic form of dilated cardiomyopathy was shown [128, 129].

To maintain abstinence, recent investigations suggest the benefits of adjuvant medications, e. g., naltrexone, which is an opiate receptor antagonist that blocks endogenous opioid reward and reduces alcohol-cue-conditioned reinforcement signals; acamprosate, an agent that exerts action through excitatory amino acids; by disulfiram, an aldehyde dehydrogenase inhibitor, which causes in alcohol use acetaldehyde accumulation and symptoms such as nausea, flushing, sweating, and tachycardia or by selective serotonin re-uptake inhibitors (SSRI) [8, 130, 131]. To treat the alcohol problem, a combined approach comprising pharmacologic and psychosocial therapy involving self-help groups or Alcoholics Anonymous is essential.

Treatment of alcoholic cardiomyopathy follows the usual regimen for therapy of heart failure, including ACE inhibitors, betablockers, diuretics including spironolactone or eplerinone, and digitalis in atrial fibrillation for rate control together with anticoagulation, whenever appropriate (Table 3). Caution for anticoagulation is warranted due to the problems of noncompliance, trauma, and overdosage especially in hepatic dysfunction.

**Table 3:** Treatment of alcoholism and alcoholic cardiomyopathy

Medication	Treatment goal	Dosage	Adverse reaction	Evidence
*Pharmacological for maintaining abstinence*				
Naltrexone	Abstinence	50–100 mg/day (oral) 380 mg i. m. per month	Nausea, headache, dizziness, joint and muscle pain	High
Acamprosate	Abstinence	666 mg three times daily	Diarhea, pruritus, rash, altered libido	High
Disulfiram	Abstinence	200 mg/day (oral)	Dizziness, rash, headache, polyneuritis, impotence, hepatotoxicity	Mixed, needs supervision
Nalmefene	Reduced drinking or abstinence	18 mg/day (oral)	Dizziness, rash, headache, nausea, vomiting	Moderate
Diazepam	Avoid delirium	As needed	Dizziness, sleepiness	Only symptomatic
*Pharmacological for heart failure (HF)*				
ACE inhibitors	HF+ prognosis	As tolerated	*–*	High in HF
Betablockers	HF+ prognosis	As tolerated	*–*	High in HF
Diuretics	HF+ prognosis	As needed	*–*	High in HF
Digitalis	Rate control	According to digoxin or digitoxin level	Avoid overdosage	Moderate in atrial fibrillation (AF)
Anticoagulants	Avoid stroke	INR 1.8–2.2 in AF	Bleeding	High in AF

## Conclusion

The individual amount of alcohol consumption decides on harm or benefit. The preponderance of data suggests that drinking one to two drinks in men and one drink in women will benefit the cardiovascular system over time. More than this amount can lead to alcoholic cardiomyopathy. Moderate drinking below that threshold might even reduce the incidence of coronary artery disease, diabetes, and heart failure.
